# A Rare Cause of Urinary Incontinence in a Child: A Case of Tarlov Cyst and a Review of Literature

**DOI:** 10.7759/cureus.67712

**Published:** 2024-08-25

**Authors:** Selin Kuzucu, Ismail Akdulum

**Affiliations:** 1 Pediatric Medicine, Sorgun State Hospital, Yozgat, TUR; 2 Radiology, Gazi University Faculty of Medicine, Ankara, TUR

**Keywords:** urinary incontinence, perineural cyst, child, inkontinans, tarlov cysts

## Abstract

Tarlov cysts are formed by ectasia of the perineural spaces around the spinal nerve roots in or distal to the dorsal root ganglion. The cerebrospinal fluid constitutes the cerebrospinal fluid content. Pathogenesis and clinical findings remain unclear. The majority of the defects are asymptomatic. However, in the case of nerve root or spinal cord compression, symptoms such as low back pain, radiculopathy, and bowel and bladder dysfunction occur.

Tarlov cyst is rare, and there is limited data on its clinical findings and incidence. This study presents a 10-year-old child patient with urinary incontinence and a Tarlov cyst. This is an interesting case due to the limited data about Tarlov cysts in pediatric patients.

## Introduction

Perineural cysts, also known as Tarlov cysts, develop between the endoneurium and perineurium of the nerve root [1,2]. They frequently occur as large dilatations of the spinal nerve root sheaths at the junction of the posterior root and dorsal ganglion [1,2]. Anatomically, they are sacs filled with cerebrospinal fluid [2]. Perineural cysts seen in the sacral roots of cadavers were first described by neurosurgeon Isadore Max Tarlov at McGill University in Canada in 1938 [1,2]. The pathogenesis of Tarlov cysts remains unclear. Many hypotheses have been proposed, including inflammation, trauma, congenital origin, and degenerative processes [1-3].

Tarlov cysts were initially considered anatomical variants of uncertain clinical significance [1]. However, there is extensive evidence that radiologically defined Tarlov cysts may be symptomatic [2,3]. Symptomatic perineural cysts are rare in pediatric patients [3]. The incidence in children is not known because they are usually asymptomatic and an MRI is required for diagnosis [3]. In this study, a pediatric patient with urinary incontinence and a Tarlov cyst will be presented.

## Case presentation

A 10-year-old female patient who was known to be healthy presented to the pediatrics outpatient clinic with a complaint of urinary incontinence during the day. It was learned that the complaint had been present for the last one year, had become more frequent for two months, and did not occur during sleep at night. It was learned that the patient had no other accompanying complaints, such as burning during urination, pain, or constipation, and had no previous urinary tract infection. During the physical examination, all system examinations were evaluated as normal.

The patient was investigated for urinary incontinence. A complete urinalysis was normal. No growth was observed in the urine culture. Blood parameters were as follows: hemoglobin: 13.3 g/Dl, leucocyte: 5.7 /L, platelet: 296.000 /L, creatinine: 0.46 mg/dl, sodium: 139 mmol/L, potassium: 4.4 mmol/L, aspartate aminotransferase: 21 U/L, alanine aminotransferase: 14 U/L, and blood glucose: 85 mg/dL were evaluated as normal (Table 1). Urinary system ultrasonography was found to be normal.

**Table 1:** Laboratory results and reference ranges of the patient AST: aspartate aminotransferase, ALT: alanine aminotransferase

	Patient’s result	Normal range
Hemoglobin	13.3 g/dL	10.1-16 g/dL
Leucocyte	5.7/L	4-10/L
Platelets	296.000/L	142.000-450.000/L
Creatinine	0.46 mg/dl	0.24-0.85 mg/dl
Sodium	139 mmol/L	135-145 mmol/L
Potassium	4.4 mmol/L	3.5-5.1 mmol/L
AST	21 U/L	0-50 U/L
ALT	14 U/L	0-50 U/L
Blood glucose	85 mg/dL	60-125 mg/dL
Urine culture	Bacterial growth did not occur	

**Figure 1:**
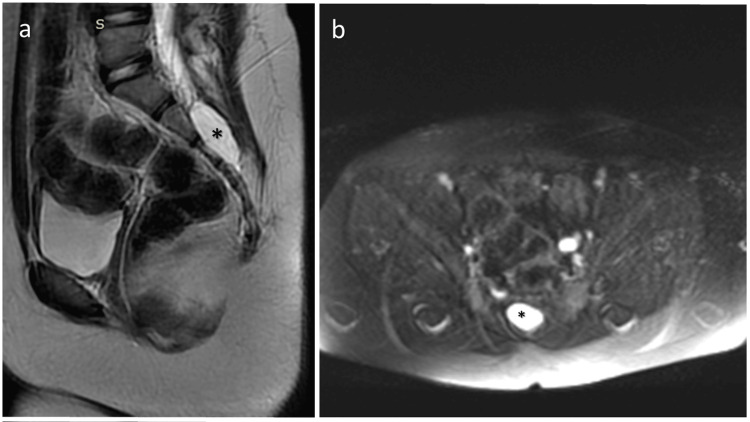
Sagittal T2 (a) and axial fat-suppressed T2 (b) MRI images showing a thin-walled Tarlov cyst measuring 43 x 17 x 23 mm located at the level of the S2-S4 vertebral corpus within the spinal canal and slightly to the right of the midline MRI: magnetic resonance imaging

Despite the patient's current imaging and laboratory tests being normal, due to the continued occurrence of daytime urinary incontinence, advanced imaging tests are planned to investigate a possible pathology that could be causing pressure. As a result, a Tarlov cyst with a diameter of approximately 4.5 cm was observed in the sacral canal (Figure 1). The patient was followed up by neurosurgery and pediatric nephrology.

## Discussion

Tarlov cysts are formed by the ectasia of the perineural spaces around the spinal nerve roots in the dorsal root ganglion or distal spinal nerve roots and consist of cerebrospinal fluid [1]. They are usually found at the sacral level. However, they can also be observed at other spinal levels [1,2]. When analyzed according to all age groups, it has been reported that the prevalence is 4.27% in the world, and it is more common in women [2]. These cysts are usually asymptomatic, and patients are diagnosed incidentally [1-3]. However, they may cause various symptoms, including low back pain, radiculopathy, bowel and bladder dysfunction, and sexual dysfunction, due to nerve root compression [1-3]. In our patient, urinary incontinence was observed as a result of Tarlov cyst compression.

Urinary incontinence is frequently encountered in childhood [4]. The most important known causes of urinary incontinence are inadequate maturation of the bladder, negative behaviors during toilet training, urinary tract infections, anatomical disorders of the lower urinary system, and neural problems. Causes related to neural pathological disorders are observed more rarely in childhood [4]. Since Tarlov cyst may be a rare neurological cause, it should be included in the differential diagnosis [3,4].

Currently, the diagnosis and treatment of these cysts are still controversial [2,3]. Although surgical excision is recommended according to the severity of clinical findings, there is uncertainty about the long-term results of surgery for these lesions in children [5]. Recurrence after surgical excision is also unknown [5].

Looking at the pediatric patients with a diagnosis of Tarlov cyst in the literature, Tarlov cyst was reported in a study conducted by Shams et al. in 2022 in a pediatric patient with a complaint of urinary incontinence and a diagnosis of Ehlers-Danlos syndrome [5]. In a study conducted by Mijalcic et al. in 2019, a Tarlov cyst was reported in a seven-year-old female patient with urinary incontinence [6]. In a study conducted by Dayyani et al. in 2019, it was reported that a Tarlov cyst was observed in an eight-month-old female patient on an MRI performed due to restlessness while in a sitting position [7]. In a study conducted by Lin et al. in 2011, it was reported that a 16-year-old female patient with radiculopathy had a Tarlov cyst [8]. In a case series conducted by Elsawaf et al. in 2016, a Tarlov cyst was reported in a seven-year-old girl and a boy with urinary incontinence [9]. In a study by Huang et al. in 2023, a Tarlov cyst was reported in a 10-year-old girl with gait disturbance [10]. In a study conducted by Siller et al. in 2024, a Tarlov cyst was reported in a 15-year-old girl with urinary incontinence and numbness in the anogenital region [11]. In a study conducted by Yoshioka et al. in 2021, a Tarlov cyst was reported in a seven-year-old girl with urinary and fecal incontinence [12]. In a study conducted by Najib et al. in 2024, a Tarlov cyst was reported in a girl with a 24-day brachial plexus injury [13].

It is interesting to note that most of the cases were girls, and most of them had complaints of urinary incontinence. Our case was also a female patient who presented with urinary incontinence. Since the literature data on the Tarlov cyst is limited, when the available data were evaluated, it was suggested that the Tarlov cyst should be considered in the differential diagnosis of female patients with urinary incontinence.

## Conclusions

Tarlov cyst is a rare pathology. Data on its clinical findings and incidence are limited. This case is of interest because of the limited data on Tarlov's cyst, especially in pediatric patients. It is important to include it in the differential diagnosis of patients with complaints such as incontinence and radiculopathy. Since the treatment of Tarlov's cyst is still controversial, there is a need for large studies to be conducted on patients with the diagnosis of Tarlov's cyst.
